# Autofluorescence and deep learning in early disease detection: biological foundations, clinical applications, and future directions

**DOI:** 10.3389/frai.2026.1859961

**Published:** 2026-06-18

**Authors:** Shunke Li, Lin Wang, Huijuan Zhao, Longgang Yu, Xudong Yan, Lin Han, Jisheng Zhang, Yan Jiang

**Affiliations:** 1 Department of Otorhinolaryngology Head and Neck Surgery, The Affiliated Hospital of Qingdao University, Qingdao, China; 2 Shandong Provincial Engineering Research Center for Precision Diagnosis and Therapy in Otorhinolaryngology, Qingdao, China; 3 Department of Child Health Care, Zibo Municipal Hospital, Zibo, China

**Keywords:** autofluorescence, deep learning, early disease detection, functional imaging biomarkers, precision medicine

## Abstract

Autofluorescence (AF) imaging enables label-free visualization of tissue metabolism and microenvironmental alterations, while deep learning (DL) provides powerful tools to decode its complex optical signatures. Their integration has emerged as a promising framework for functional and biologically informed disease assessment. Recent studies demonstrate that AF-DL approaches improve lesion detection, intraoperative guidance, and early therapeutic response evaluation across multiple organ systems. By leveraging multidimensional spectral, temporal, and spatial features, DL mitigates the intrinsic variability and limited specificity of standalone AF imaging. This review summarizes current diagnostic, prognostic, and surgical applications of AF-DL integration, with particular emphasis on model interpretability, generalizability, and biological relevance. Key challenges, including device dependence, dataset heterogeneity, annotation burden, and regulatory considerations, are critically discussed. Finally, future directions are proposed toward standardized acquisition, prospective multicenter validation, and clinically integrated workflows. By bridging intrinsic tissue biochemistry with data-driven intelligence, AF-DL integration offers a new class of functional imaging biomarkers with significant potential for precision diagnosis, surgery, and treatment monitoring.

## 1 Introduction

Early disease detection remains a central challenge and opportunity in modern medicine. Across oncology, metabolic disease, neurodegeneration, ophthalmology, and inflammatory disorders, the earliest pathological changes frequently begin as biochemical and metabolic alterations long before morphological abnormalities can be detected by conventional imaging or histopathology (Miotto et al., 2018). These latent metabolic disturbances often determine disease trajectory, therapeutic responsiveness, and long-term outcomes. Thus, identifying these early alterations represents a critical step toward preventing disease progression and enabling truly precision-based, preemptive clinical interventions (García-Plazaola et al., 2015).

Autofluorescence (AF) imaging has emerged as a uniquely valuable modality for early diagnostics because it enables label-free, real-time quantification of endogenous fluorophores such as reduced nicotinamide adenine dinucleotide (NADH), Flavin adenine dinucleotide (FAD), collagen, elastin, lipofuscin, porphyrins, and advanced glycation end products (AGEs). Providing direct insights into cellular metabolism, redox status, mitochondrial function, extracellular matrix (ECM) remodeling, and oxidative stress (Croce and Bottiroli, 2014). These biochemical pathways represent some of the earliest hallmarks of diseases such as dysplasia, diabetes, and chronic inflammation (Mukunda et al., 2022). Unlike contrast-based imaging, AF interrogates tissues without external dyes, enabling intraoperative, longitudinal, and bedside metabolic assessments with minimal perturbation (Zhou J. et al., 2024).

Simultaneously, deep learning (DL) has revolutionized biomedical imaging by uncovering nonlinear spatial, spectral, and temporal patterns that exceed human visual perception. Convolutional neural networks, attention-based architectures, and multimodal fusion models have demonstrated strong performance in identifying subtle pathological features, predicting molecular alterations, stratifying risk, and guiding clinical decision-making in radiology, pathology, ophthalmology, endoscopy, and surgery (Chen X. et al., 2022). The expanding availability of digital imaging and large-scale medical datasets has accelerated the development of intelligent diagnostic systems across specialties (Puttagunta and Ravi, 2021).

The integration of AF with DL represents a paradigm shift toward next-generation early diagnostics. AF provides high-resolution biochemical contrast, while DL transforms these signals into actionable clinical predictions. Emerging evidence suggests that this synergy may enable: (1) detection of pre-morphological metabolic abnormalities (Coda et al., 2014); (2) differentiation of benign, inflammatory, and precancerous lesions (Sawan and Mashlah, 2015; Warke and Khot, 2021); (3) prediction of treatment response and disease progression (Rauch et al., 2023); (4) precision surgical navigation and organ-preserving interventions (Shi et al., 2024); and (5) development of virtual metabolic histology platforms (Wong P. F. et al., 2024).

This combined AF-DL framework enables transition from structural to metabolic and molecular-level diagnostics, offering exceptional sensitivity in diseases characterized by early metabolic rewiring, including cancer, diabetes, chronic inflammatory disorders, and neurodegenerative diseases (van Waateringe et al., 2019). The integration of metabolic AF signatures with Artificial Intelligence (AI) also allows detection of subtle biochemical alterations invisible to standard imaging systems (Duran-Sierra et al., 2020).

However, several challenges remain. AF signals demonstrate variability across tissue types, patient demographics, devices, and excitation methods (Wizenty et al., 2020). DL models face issues related to data bias, interpretability, reproducibility, and generalizability across institutions (Xu et al., 2024). Promising solutions include hyperspectral AF, fluorescence lifetime imaging (FLIM), multimodal fusion networks, and emerging foundation models that leverage cross-domain biomedical data (Wang et al., 2024).

In this review, we integrate biological principles of AF, computational foundations of DL, and rapidly expanding AF-DL applications across major medical specialties. We outline key technological innovations, clinical translation pathways, mechanistic insights, and emerging trends that will shape future intelligent diagnostic systems. Our goal is to establish a unified framework for understanding and advancing AF-guided, AI-driven early disease detection.

## 2 Literature search strategy and selection criteria

We searched the PubMed, IEEE Xplore, and Web of Science databases for relevant articles and conducted a literature search using the following keywords: “autofluorescence imaging,” “fluorescence lifetime imaging,” “reduced nicotinamide adenine dinucleotide,” “flavin adenine dinucleotide,” “endogenous fluorophores,” “collagen autofluorescence,” “lipofuscin,” “porphyrins,” “deep learning,” “convolutional neural network,” “machine learning,” “artificial intelligence,” “transfer learning,” and “generative adversarial network.” All keywords were used in all possible permutations and the abstracts of the search results were evaluated. No date limit was set for this review. Older articles were identified manually by searching the reference lists of articles that satisfied the inclusion criteria. Secondary sources from the reference lists of the selected primary papers were searched, evaluated for appropriateness, and included when appropriate.

**Figure 1:**
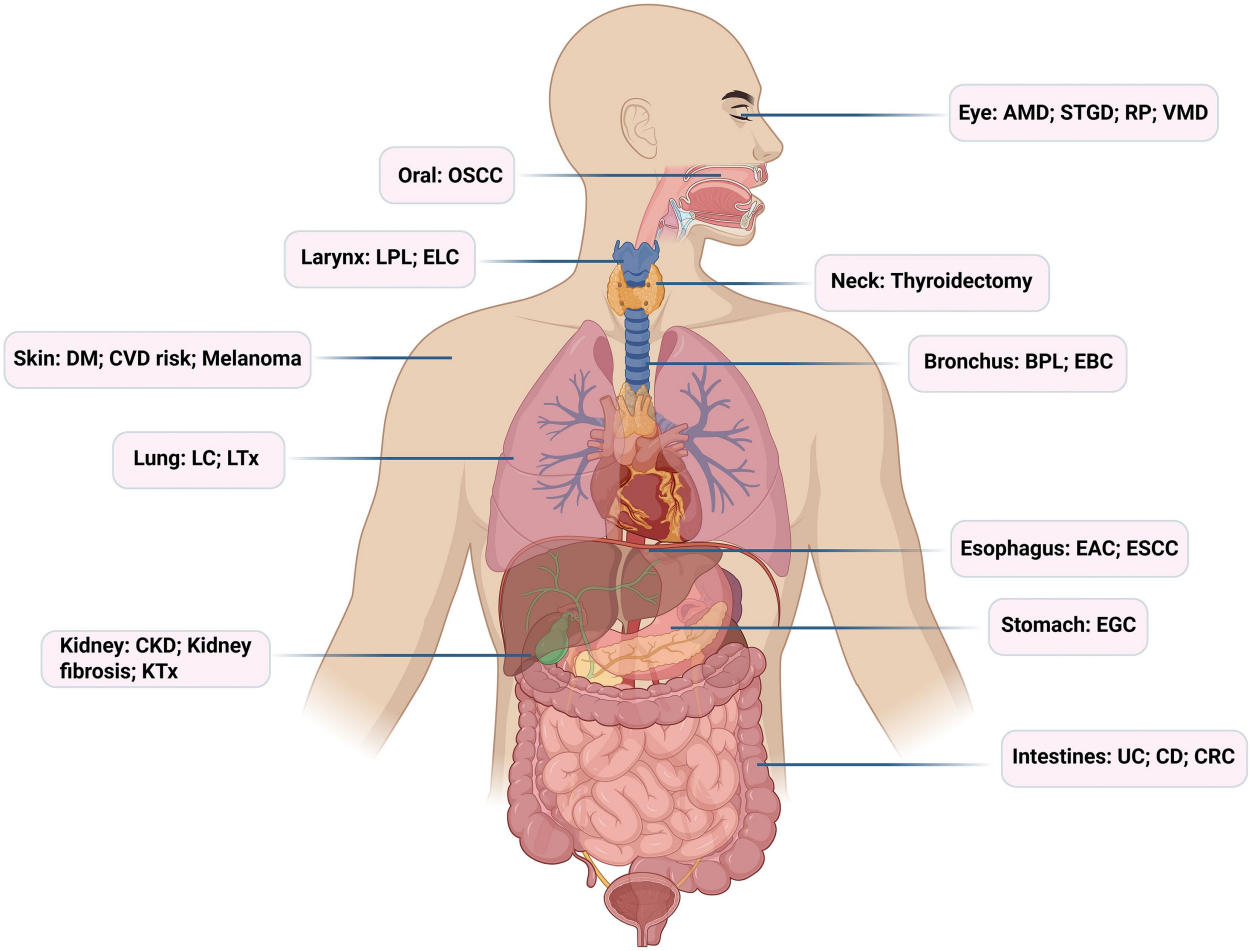
Clinically established applications of autofluorescence-based diagnosis across organ systems. Abbreviations: AMD, age-related macular degeneration; STGD, Stargardt disease; RP, retinitis pigmentosa; VMD, vitelliform macular dystrophy; OSCC, oral squamous cell carcinoma; LPL, laryngeal premalignant lesions; ELC, early laryngeal carcinoma; DM, diabetes mellitus; CVD, cardiovascular disease; LC, lung cancer; LTx, lung transplantation; CKD, chronic kidney disease; KTx, kidney transplantation; BPL, bronchial premalignant lesions; EBC, early bronchial cancer; EAC, esophageal adenocarcinoma; ESCC, esophageal squamous cell carcinoma; EGC, early gastric cancer; UC, ulcerative colitis; CD, Crohn’s disease; CRC, colorectal cancer (Created with BioRender).

An overview of representative autofluorescence-based applications across different organ systems is shown in Figure 1.

## 3 Biological and computational foundations

### 3.1 Biological basis of AF

AF arises from endogenous biomolecules capable of absorbing photons and emitting fluorescence at longer wavelengths. These fluorophores reflect key metabolic and structural characteristics, enabling label-free visualization of cellular redox reactions, mitochondrial activity, ECM composition, and biochemical remodeling (Croce and Bottiroli, 2014). In healthy tissues, AF intensity and spectra remain tightly regulated, representing stable biochemical states. However, disease alters concentrations, redox ratios, and microenvironmental context of fluorophores, producing distinct spectral fingerprints long before morphological changes develop (Varone et al., 2014; Chen L. et al., 2023). Redox cofactors such as NADH and FAD serve as optical surrogates for metabolic programming, and their detailed spectral properties and diagnostic implications are elaborated in Section 3.2 (Gopalakrishnan et al., 2020; Siqueira et al., 2024; Guo et al., 2025). AF also originates from collagen cross-links and elastin fibers, providing structural information about tissue integrity and ECM remodeling in relation to disease progression and enabling truly precision-based, preemptive clinical interventions (García-Plazaola et al., 2015; Meleshina et al., 2018; Nongnuch and Davenport, 2015). These biological insights underscore why AF is inherently sensitive to early disease processes: metabolic imbalance and extracellular remodeling occur far earlier than morphologic lesions detectable by conventional imaging.

### 3.2 Major endogenous fluorophores and their diagnostic implications

AF in biological tissues originates from a distinct set of endogenous chromophores whose unique spectral signatures and microenvironmental sensitivity provide complementary insights into metabolism, structural integrity, and cumulative biochemical stress (Wizenty et al., 2020).

The primary sources of metabolically informative AF are the redox cofactors reduced nicotinamide adenine dinucleotide (NADH/NADPH, often measured collectively as NAD(P)H) and FAD. NAD(P)H fluorescence, predominantly from its reduced form, serves as an optical reporter of the balance between glycolysis and oxidative phosphorylation. Variations in its intensity and fluorescence lifetime reflect shifts in mitochondrial function, hypoxia, and the cellular balance between anabolic and catabolic states (Lukina et al., 2019). FAD fluoresces in the green spectral region and acts as the oxidized counterpart in redox pairs (Theodossiou et al., 2024). Integrating signals from NAD(P)H and FAD to form optical redox ratios significantly enhances the discrimination between glycolytic and oxidative phenotypes, while improving robustness against single-channel variability (Zhou et al., 2020). This approach is particularly powerful because early stages of cancer, inflammation, and ischemic injury are characterized by metabolic reprogramming. Consequently, NAD(P)H-FAD imaging, especially when combined with fluorescence lifetime measurements (FLIM), has become a sensitive tool for detecting pathology before morphological changes become apparent (Zhou H. et al., 2024).

Beyond metabolic cofactors, structural proteins like collagen and elastin are major sources of AF signals, reporting on the architecture and remodeling of the ECM (Giovannacci et al., 2019). Collagen cross-links and elastin fibers dominate the AF signal in connective tissues. Under multiphoton excitation, these structures also generate second-harmonic generation (SHG) contrast, enabling depth-resolved assessment of stromal organization. Alterations in fiber density, alignment, or cross-linking that are detectable via combined AF/SHG imaging are correlated with fibrotic progression, the desmoplastic reaction in tumors, and mucosal remodeling in chronic inflammatory conditions (Klinger et al., 2012). Critically, structural AF provides context for metabolic AF, as stromal changes often precede or accompany epithelial metabolic rewiring. An integrated analysis of both signal types is therefore essential for accurate phenotyping in early disease.

Several other fluorophores with relevance to specific tissues or diseases are also diagnostically important. In the retina, bisretinoid-derived lipofuscin accumulates in the retinal pigment epithelium (RPE), generating the dominant signal in fundus autofluorescence (FAF) imaging. Spatial patterns and quantitative FAF metrics are well-validated biomarkers for monitoring RPE stress and photoreceptor turnover in inherited retinal dystrophies and age-related macular degeneration (Miere et al., 2020; Obana et al., 2024). AGEs accumulate over time in long-lived proteins, producing a long-lived fluorescence signal measurable in skin and vascular tissues. This signal provides an integrated index of cumulative metabolic stress, proving valuable for risk stratification in diabetes and chronic kidney disease (van Waateringe et al., 2019; Yozgatli et al., 2018; Tanaka et al., 2013). Porphyrins, which can be of host or microbial origin, emit at longer wavelengths and often highlight dysbiotic or dysplastic mucosal areas, a feature exploited by several clinical fluorescence-guided devices (Black et al., 2021).

The diagnostic utility of each fluorophore class is highly dependent on the acquisition strategy and interpretive framework. Simple intensity-based measurements, while clinically convenient, are susceptible to confounding factors like uneven illumination, light scattering, absorption, and media opacities. In contrast, advanced techniques such as multispectral imaging and FLIM can separate overlapping emissions and distinguish between bound and free states of metabolic cofactors, thereby substantially improving diagnostic specificity (Lukina et al., 2019; Pham et al., 2025). The spatial scale of analysis is equally critical: subcellular FLIM can reveal early mitochondrial dysfunction, while wide-field multispectral AF is better suited for screening and intraoperative mapping (Qian et al., 2022). Therefore, optimal AF protocols typically combine spectral, temporal, and spatial modalities, supported by standardized calibration using phantoms and reference spectra, alongside rigorous preprocessing to compensate for background and scattering.

From a translational standpoint, the complementary roles of different fluorophores advocate for tailored applications rather than a universal assay. NAD(P)H-FAD redox imaging excels in detecting early epithelial dysplasia and monitoring rapid pharmacodynamic responses (Eggert et al., 2025). Collagen/SHG imaging is more informative for characterizing fibrotic diseases and tumor stroma. Lipofuscin-based FAF is indispensable for retinal disease management (Obaid et al., 2022), and skin AGE measurement offers a tool for population-level metabolic risk stratification (Chen J. et al., 2023). Recognizing these modality-specific strengths guides targeted clinical deployment and informs the design of DL models, which should be adapted to the dominant signal type (metabolic, structural, or composite) and the specific clinical objective.

Finally, successful clinical implementation requires careful consideration of pre-analytical and instrument-dependent variables that can distort the biological signal. Factors such as skin pigmentation, anterior media opacities (e.g., cataracts), probe-tissue contact pressure, and inter-device variability all influence AF measurements (Momma et al., 2012; Sharifzadeh et al., 2014). Robust translation therefore necessitates device harmonization (Turski et al., 2020), validation across diverse populations, and the inclusion of these covariates during model training to prevent biased or non-generalizable classifiers. When these technical and biological challenges are systematically addressed, AF-derived biomarkers evolve into powerful, mechanistically interpretable tools for early disease detection.

### 3.3 Optical and biophysical mechanisms of AF signals

The effective acquisition and interpretation of AF signals rely on a thorough understanding of their underlying optical and biophysical mechanisms.

Excitation-Emission Spectral Features and Tissue Optical Properties: Each endogenous fluorophore possesses unique excitation and emission spectra, which form the basis for spectral identification. For instance, NAD(P)H has a peak excitation around 350 nm and emits around 450 nm (blue), whereas FAD is excited around 450 nm and emits around 525 nm (green) (Dysli et al., 2017). However, *in vivo* imaging is complicated by significant signal distortion due to tissue scattering and absorption. The strong absorption of hemoglobin in the blue-green spectrum (400–500 nm) can markedly attenuate AF signals from superficial vascularized regions or deeper tissues, leading to underestimation (Olivo et al., 2012). Similarly, tissue scattering limits excitation light penetration and causes spatial dispersion of emitted light, reducing image resolution and signal-to-noise ratio (Zheng et al., 2021). Consequently, any quantitative AF analysis must account for these intrinsic optical filter effects, or alternatively, utilize excitation windows in the near-infrared spectrum to avoid regions of high absorption.

Modulation of Fluorescence Signals by Microenvironment and Pathophysiology: AF signals reflect not only fluorophore concentration but also act as sensitive probes of their molecular microenvironment. Hypoxia, by inhibiting the mitochondrial electron transport chain, leads to NAD(P)H accumulation, increasing its fluorescence intensity and potentially altering its enzyme-bound ratio, which in turn affects its fluorescence lifetime (Bickler et al., 2009). ECM remodeling, such as increased collagen cross-linking in fibrosis, enhances the AF from structural proteins while simultaneously altering tissue scattering properties, indirectly affecting the detection of metabolic fluorophores (Leblanc et al., 2025). Oxidative stress can directly quench certain fluorophores (e.g., FAD) or induce the accumulation of senescence-associated fluorophores like lipofuscin, permanently altering the tissue’s AF fingerprint. Understanding these regulatory mechanisms is crucial for linking AF changes to specific pathobiological processes.

Comparison of AF Imaging Modalities: Various AF imaging modalities have been developed, each suited to different scientific and clinical questions. Wide-field AF offers the simplest and fastest approach, making it suitable for large-area screening and real-time intraoperative navigation; its primary limitation is the provision of only intensity information, resulting in lower specificity (Sawan and Mashlah, 2015; Li et al., 2022). Multispectral AF acquires signals at several discrete spectral bands, partially separating overlapping emissions and improving discrimination between fluorophores (Aihara et al., 2012; Osada et al., 2011). Hyperspectral AF provides continuous spectral resolution, enabling fine differentiation of fluorophores with highly overlapping spectra, though large data volumes and slow acquisition speeds currently limit real-time clinical application (Collins et al., 2022). FLIM measures the decay time of fluorescence after excitation, which is highly sensitive to the fluorophore’s molecular environment (e.g., binding state, pH, temperature) but largely independent of concentration and excitation intensity. For example, free and enzyme-bound NAD(P)H have distinct lifetimes, allowing FLIM to non-invasively quantify cellular metabolic states (Ouyang et al., 2021). FLIM thus provides functional information inaccessible to conventional intensity-based imaging, albeit with more complex, costly systems and typically slower data acquisition rates.

### 3.4 Computational foundations of DL for AF analysis

The powerful pattern recognition capabilities of DL enable the extraction of subtle features from high-dimensional, complex AF data that surpass human visual perception. The choice of model architecture is closely tied to the characteristics of the data.

Evolution and Adaptation of Core Network Architectures: In AF image analysis, Convolutional Neural Networks (CNNs) and their variants (e.g., U-Net, DeepLab) remain the workhorses for spatial feature extraction, excelling at segmenting lesions or identifying specific morphological patterns from wide-field or multispectral AF images (Li M. et al., 2023). Transformer architectures, with their strong global context modeling capabilities, show promise for tasks requiring long-range dependencies or for sequential data. For tasks where multi-scale information is critical (e.g., from cellular metabolic anomalies to tissue-level structural disruption), multi-scale convolutional architectures or Feature Pyramid Networks (FPNs) have proven more effective (Huang T. et al., 2025).

Specialized Modeling Strategies for AF Data Characteristics: Spectral-Spatial Dual-Channel Modeling: For multispectral or hyperspectral AF data, leveraging both spatial context and spectral features is essential. Common strategies include: early fusion (stacking multiple bands as multi-channel input); late fusion (extracting spatial and spectral features with separate network branches before fusion); and more complex 3D convolutions or spectral attention mechanisms that directly process the data cube (Jansen-Winkeln et al., 2021).

DL Models Specific to FLIM: FLIM data typically exist as per-pixel time-decay curves or fitted parameter maps (e.g., mean lifetime, component fractions). Processing such data may involve using 1D CNNs or long short-term memory for decay curves, or 2D CNNs for parameter maps. More advanced methods perform end-to-end analysis on raw photon-counting data to avoid information loss during the fitting process (Li J. et al., 2025).

Multimodal Fusion: Integrating AF information with other imaging modalities, enables complementary advantages. DL facilitates pixel-level feature alignment and deep fusion (Roney et al., 2021). For instance, AF can highlight areas of metabolic abnormality, which can then be confirmed for structural damage by optical coherence tomography (OCT), significantly boosting diagnostic confidence (Zhu et al., 2024).

Interpretability and Model Reliability: The “black-box” nature of DL models is a major obstacle to their clinical adoption (Singh et al., 2025). Interpretability tools (e.g., Grad-CAM, SHAP) can visualize the image regions or key spectral bands upon which a model bases its decisions. This is crucial for building clinician trust and potentially discovering new biomarkers (Nawaz et al., 2025; Ye et al., 2024; Feng et al., 2025). Simultaneously, common challenges in DL must be addressed: Data bias (performance degradation due to variations across devices or populations) necessitates training on diverse datasets and employing standardization techniques (Zhu et al., 2022). Overfitting and the small sample size problem are particularly acute in medicine, requiring strategies like transfer learning, data augmentation using Generative Adversarial Networks (GANs), and few-shot learning to mitigate them (Muchuchuti and Viriri, 2023; Huang and Shu, 2025).

## 4 Organ-system-based clinical applications

This section provides a comprehensive, critical review of the integrated AF-DL framework across medical specialties, evaluating its evidence, clinical impact, and persistent challenges. The major clinical applications of AF-DL integration across different organ systems are summarized in Table 1.

**Table 1:** Clinical applications of AF-DL for diagnosis and tissue characterization.

Scenario	Clinical task	Disease /target	AF modality	Endogenous AF target(s)	Excitation (wavelength, nm)	DL model	References	Study type	AF alone	AF-DL	Added value
Ophthalmology	Classification	Inherited retinal diseases	FAF	Lipofuscin	500–800	CNN	Miere et al. (2020)	Retrospective clinical validation	expert-dependent	Accuracy 95.0%, AUC > 0.995	Automated classification, reduced expert reliance
Head and neck oncology	Detection	HNSCC	AFI	Not reported	455	CNN	Halicek et al. (2019)	Prospective ex vivo clinical study	AUC = 0.73	AUC = 0.85–0.95	Higher AUC, outperforms dyes
Head and neck oncology	Tissue identification	Parathyroid gland during thyroidectomy	NIR-AFI	Not reported	785	Google AutoML Vision	Avci et al. (2022a)	Prospective diagnostic clinical study	Subjective interpretation, prone to interference	Precision 95.7%	Shortens learning curve, reduces false positives
Oral cavity	Detection	Oral premalignant	AF-FLIM	Collagen, NADH, FAD, porphyrins	355	SVM/RF/CNN	Marsden et al. (2021)	Prospective ex vivo diagnostic clinical study	limited diagnostic accuracy	Accuracy 92.3%	Improved accuracy, reduced inter-observer variability
Colon	Detection	Colorectal cancer	HSAFI	Not reported	360–555	CihanNet/ResNet50	Oswald et al. (2024)	Retrospective ex vivo diagnostic study	low diagnostic accuracy	Accuracy 93.0%	Higher accuracy, real-time interpretable
Kidney	Classification	CKD	MSAFI	NAD(P)H; FAD	340–510	ML	Mahbub et al. (2021)	Prospective clinical diagnostic study	AUC = 0.75–0.85	AUC = 0.90–0.99	Non-invasive, high-precision detection
Kidney transplant	Differential diagnosis	KTx graft dysfunction	MSAFI	NAD(P)H; FAD (cell metabolic features)	340–510	ML	Wu et al. (2025)	Prospective clinical diagnostic study	Subjective visual inspection only	AUC = 0.91–0.95	Differentiates graft dysfunction causes
Skin oncology	Pixel-level classification	Pigmented skin lesions	maFLIM	Collagen, NADH, FAD	355	DNN	Vasanthakumari et al. (2024)	Prospective clinical diagnostic study	Accuracy 60%	AUC = 0.88	Boosts accuracy, pixel-level lesion mapping
Dermatology	Diagnosis support	Melanoma	maFLIM	NADH, FAD	340–510	ML	Knab et al. (2024)	Ex vivo cell-based diagnostic study	Low diagnostic accuracy	AUC = 0.928	Enhances accuracy, objective classification
Pulmonology	Detection	lung cancer	AFB	Not reported	395–445	ESFPNet	Winiarski et al. (2025)	Retrospective and prospective diagnostic studies	Accuracy 60–80%	AUC = 0.97–0.98	Reduces variability, improves detection accuracy
Hematology	Classification	5-part leukocyte differential	Smart-AM	Hemoglobin; NADH; aromatic amino acids	265–270	Mask R-CNN variants	Huang et al. (2024)	Prospective ex vivo diagnostic study	Subjective visualization, low efficiency	Accuracy 0.982	Auto-classification, virtual staining, time-saving
Digital pathology	Virtual H&E staining	Tissue virtual staining	AF-FLIM	Multiple endogenous fluorophores	485	cGAN	Wang et al. (2024)	Retrospective ex vivo diagnostic study	MSSIM 0.53–0.69, no cellular-level interpretation	MSSIM 0.53–0.70, PSNR 20.20–23.03	Virtual H&E staining, cellular lifetime identification

AF, autofluorescence; DL, deep learning; FAF, fundus autofluorescence; CNN, convolutional neural network; HNSCC, head and neck squamous cell carcinoma; AFI, autofluorescence imaging; AUC, Area Under the Curve; NIR-AFI, near-infrared autofluorescence imaging; AF-FLIM, autofluorescence fluorescence lifetime imaging microscopy; NADH, nicotinamide adenine dinucleotide, reduced form; FAD, flavin adenine dinucleotide; SVM, support vector machine; RF, random forest; HSAFI, hyperspectral autofluorescence imaging; CKD, chronic kidney disease; MSAFI, multispectral autofluorescence imaging; NAD(P)H, nicotinamide adenine dinucleotide phosphate, reduced form; ML, machine learning; KTx, kidney transplantation; maFLIM, multispectral autofluorescence lifetime imaging; DNN, deep neural network; AFB, autofluorescence bronchoscopy; Smart-AM, Smart Autofluorescence Microscopy; H&E, Hematoxylin and Eosin; cGAN, conditional generative adversarial network; MSSIM, Mean Structural Similarity Index Measure; PSNR, Peak Signal-to-Noise Ratio.

### 4.1 Head and neck system

The head and neck region, with its diverse, accessible mucosal surfaces and critical structures, is a prime territory for AF-DL applications.

#### 4.1.1 Ophthalmology

FAF imaging is a cornerstone for managing retinal diseases. Quantitative AF (qAF) aims to standardize measurements, correcting for lens opacity and ocular media density, enabling longitudinal tracking of disease progression in conditions like age-related macular degeneration (AMD) and Stargardt disease (Jolly et al., 2016; Deitch et al., 2021). DL models have achieved high accuracy in classifying hereditary retinal dystrophies (e.g., retinitis pigmentosa, vitelliform macular dystrophy) based on FAF patterns (Miere et al., 2020). Beyond diagnosis, DL applied to serial FAF images shows promise in predicting the growth rate of geographic atrophy (GA) in AMD, offering a tool for personalized prognosis and therapeutic trial monitoring. These models extract subtle patterns RPE disruption that precede visible lesion expansion (Rodríguez et al., 2020; Ong et al., 2025). Thus, FAF-based classification of inherited retinal diseases is now clinically established, whereas DL-driven GA progression prediction remains an emerging application requiring further validation.

#### 4.1.2 Thyroid and parathyroid surgery

Near-infrared AF (NIR-AF) has revolutionized parathyroid gland (PG) identification during thyroidectomy. PGs exhibit intense AF (peak ~820 nm) due to high concentrations of oxyhemoglobin and other fluorophores, distinct from thyroid and lymph nodes (Akgun et al., 2024; McEntee et al., 2024). DL models, such as CNNs, process real-time NIR-AF video streams to automatically localize PGs, often with accuracy exceeding 97%, and even predict gland viability by analyzing signal intensity patterns (Avci et al., 2022a,b). Kose et al. confirmed that DL-aided AF guidance, which achieves 98.5% sensitivity and 97.2% specificity in distinguishing parathyroid tissue, can effectively reduce the incidence of postoperative transient and permanent hypoparathyroidism. This strategy also shortens operative time and markedly flattens the learning curve for endocrine surgeons (Kose et al., 2020). Overall, DL-assisted NIR-AF for parathyroid identification is now entering routine clinical use in high-volume endocrine surgery centers, supported by consistent prospective evidence.

#### 4.1.3 Oral and oropharyngeal neoplasia

While devices like VELscope (blue-light AF) are used for oral cancer screening, they suffer from high false-positive rates due to inflammation (Wang et al., 2022; Moro et al., 2010). Multispectral AF, capturing a broader spectral range, provides more specific information. DL algorithms trained on multispectral AF images can differentiate benign inflammatory lesions from true dysplastic and malignant changes with superior specificity. Furthermore, integrated AF-DL systems are being explored for intraoperative margin assessment. Relevant studies confirmed that real-time analysis of AF spectral characteristics at surgical resection margins can help surgeons identify suspicious invasive margins efficiently. This label-free fluorescence lifetime imaging method achieves a tissue discrimination Area Under the Curve (AUC) value of 0.88, which can effectively reduce positive surgical margin rates and minimize the demand for secondary re-excision (Marsden et al., 2021). A large-scale study of 102 patients with head and neck squamous cell carcinoma also demonstrated that AF imaging based on reflectance hyperspectral imaging combined with deep learning algorithms achieved high accuracy (AUC > 0.85) in tumor margin detection, significantly outperforming fluorescent dye-based methods (Halicek et al., 2019). While wide-field AF screening tools are already clinically deployed, AF-DL margin assessment and lesion discrimination remain investigational, with validation predominantly from single center studies.

#### 4.1.4 Nasal and paranasal sinus diseases

AF endoscopy has been investigated as an adjunct optical modality for mucosal lesion detection across the upper aerodigestive tract, where altered AF patterns may reveal epithelial dysplasia or early malignant change beyond conventional white-light inspection. However, sinonasal-specific evidence for AF-based endotyping or outcome prediction remains limited, and most fluorescence-enabled advances in endoscopic sinonasal/skull-base surgery to date rely on exogenous fluorescence-guided strategies rather than endogenous AF (Mat Lazim et al., 2022; Hart et al., 2020; Suero Molina et al., 2024).

For chronic rhinosinusitis (CRS), the field is rapidly moving toward endotype-driven precision care, especially for type 2 inflammation where biologics are increasingly used (Wang et al., 2025); contemporary consensus and recent reviews highlight both the promise and practical limitations of endotyping in routine practice and underscore the need for noninvasive biomarkers (Toppila-Salmi et al., 2025). In this context, AF/FLIM represents a biologically plausible, label-free readout of metabolic state and ECM remodeling, and DL could provide robust quantification of subtle spectral-spatial features from endoscopic imaging (Ma et al., 2022).

#### 4.1.5 Larynx and hypopharynx

AF laryngoscopy (AFL) has long been explored as an adjunct to white-light endoscopy for early detection and delineation of laryngeal premalignant lesions and microinvasive carcinoma, typically presenting as loss of green stromal fluorescence related to collagen alteration and epithelial changes. Early clinical studies demonstrated that AFL could improve visualization of lesion extent compared with conventional inspection, while also highlighting typical pitfalls such as inflammation, scarring, and marked hyperkeratosis (Fostiropoulos et al., 2016).

More recently, systematic evidence continues to support AFL as a useful complementary modality with high diagnostic value for early laryngeal carcinoma and precancerous lesions, though heterogeneity in devices, thresholds, and reference standards remains a key limitation for widespread standardization (Ge et al., 2021).

From an “AF-DL” perspective, DL is particularly impactful in the larynx/hypopharynx because video streams are susceptible to motion, specular reflection, and illumination variation: DL-based quality control, semantic segmentation of vocal fold subregions, and attention-driven classification can reduce false positives driven by inflammation/scarring and provide saliency maps for interpretability, an approach consistent with broader UADT AF guidance (Mat Lazim et al., 2022). AFL is thus considered a clinically useful adjunct rather than a standalone diagnostic, and its DL-enhanced interpretation is still at an early investigational stage.

### 4.2 Respiratory system

#### 4.2.1 Lung cancer screening and bronchoscopy

AF bronchoscopy (AFB) enhances the contrast of premalignant and early malignant bronchial mucosal changes relative to white-light bronchoscopy (WLB), typically improving sensitivity but at the cost of reduced specificity (e.g., inflammation-related false positives). A meta-analysis verified that AF bronchoscopy achieves a pooled sensitivity of 0.92 for bronchial cancer detection, significantly higher than 0.70 of white-light bronchoscopy. Such high sensitivity helps lower clinical missed diagnosis rates, though this technique is more suitable for targeted examination instead of general population screening (Sun et al., 2020).

Randomized evidence also supports that WLB + AFB can outperform WLB alone for detecting preneoplastic lesions, while still cautioning against indiscriminate screening use, consistent with current practice where AFB is mainly applied to high-risk groups or for targeting biopsy in suspicious mucosal abnormalities (Häussinger et al., 2005). From an “AF-DL” perspective, recent reviews summarize emerging DL pipelines for AFB image analysis, including CNN-based approaches for lesion recognition/segmentation, and highlight ESFPNet as a representative DL model reported for AFB-related lesion analysis (Winiarski et al., 2025). While AFB itself is clinically validated for high-risk groups, DL-based interpretation of AFB images remains an emerging technology not yet integrated into routine bronchoscopy workflows.

#### 4.2.2 Airway complications post-lung transplantation

AFB is sensitive to early ischemic changes in bronchial mucosa following lung transplantation. A decline in the AF red/green ratio at the anastomotic site has been correlated with subsequent development of stenosis or dehiscence (Mendogni et al., 2020). DL can quantify these subtle spectral ratio changes over time from serial AFB videos, providing a risk score for complication development. This enables pre-emptive interventions, such as intensified immunosuppression or bronchoscopic balloon dilation, before critical stenosis occurs. This application is currently investigational, with proof-of-concept data awaiting prospective validation.

#### 4.2.3 Chronic inflammatory airway diseases

In diseases like asthma and chronic obstructive pulmonary disease (COPD), airway remodeling is a key pathological feature. AF imaging of bronchial mucosa, sensitive to collagen and elastin changes, can provide a label-free measure of subepithelial fibrosis. DL analysis can quantify this “fibrosis signal” from AF images, offering a potential endpoint for clinical trials of anti-fibrotic therapies and a tool for phenotyping patients with remodeling-dominant disease. This remains a preclinical and early translational concept.

### 4.3 Digestive system

The gastrointestinal tract, with its extensive mucosal lining and high incidence of inflammatory and neoplastic diseases, represents a prime application field for endoscopic AF imaging. The integration of DL has begun to transform AF from a qualitative adjunct into a quantitative diagnostic tool.

#### 4.3.1 Barrett’s esophagus

In Barrett’s esophagus surveillance, the endoscopic tri-modal imaging approach (white-light, narrow-band imaging, and AF imaging) has been shown to improve the detection of high-grade intraepithelial neoplasia (Curvers et al., 2010). AF imaging in this context visualizes areas of suspicious fluorescence (e.g., violet/blue), often corresponding to metabolic and architectural changes in dysplastic tissue. DL models are being developed to standardize the interpretation of these complex image sets. For instance, CNNs can analyze AF imaging patterns to automatically grade dysplasia, potentially reducing inter-observer variability among endoscopists. These models learn to associate specific spectral-shift patterns with histological grade, focusing not just on morphology but on the early metabolic shifts that precede visible morphological change. AF imaging is clinically integrated in trimodal endoscopy platforms, while DL-based dysplasia grading is still under active investigation.

#### 4.3.2 Colorectal neoplasia

AF colonoscopy enhances the detection of flat and depressed lesions, which are easily missed under white light. Dysplastic adenomas and carcinomas often exhibit decreased green AF intensity compared to surrounding normal mucosa. Advanced hyperspectral AF imaging systems capture the full emission spectrum at each point. Oswald et al. demonstrated that fluorescence excitation-scanning hyperspectral imaging, when integrated with scalable 2D-3D deep learning frameworks, supports real-time *in vivo* classification of colorectal lesions during colonoscopy, accompanied by visualized model interpretability and adjustable speed accuracy tradeoffs for clinical implementation (Oswald et al., 2024). Furthermore, in endoscopic mucosal resection (EMR) and submucosal dissection, real-time AF-DL analysis of the resection margin can provide feedback on potential residual neoplastic tissue, aiming to achieve complete en-bloc resection rates. AF-DL polyp classification is approaching clinical translation, though multicenter validation remains limited.

#### 4.3.3 Gastric and early Esophageal squamous cell carcinoma

Similar to other mucosal surfaces, early gastric and esophageal cancers display altered AF signatures. Research focuses on using multispectral AF endoscopy to capture the redox state (via NAD(P)H/FAD ratio) and stromal collagen changes. DL algorithms, such as multi-scale fusion CNNs, trained on paired AF images and histology, can identify early cancerous foci and guide targeted biopsies. A recent study demonstrated that a DL model analyzing AF imaging could predict the invasion depth of early gastric cancer, which is critical for deciding between endoscopic and surgical treatment (Wu et al., 2022). This exemplifies the progression from detection to prognostic stratification. More recently, advancements in the second near-infrared window (NIR-II, 1000–1700 nm) have further expanded the clinical utility of AF imaging. He et al. (2026) successfully utilized NIR-II AF for the label-free visualization of human liver malignancies, achieving significantly higher tumor-to-normal tissue contrast and deeper penetration compared to traditional visible-light imaging. This progress underscores the evolving capability of AF systems in achieving molecular-level precision in complex surgical environments. These applications, including NIR-II AF, are currently at the translational research stage, with initial clinical feasibility demonstrated but not yet standard practice.

#### 4.3.4 Inflammatory bowel disease (IBD)

Quantitative AF imaging offers a novel, label-free method to grade mucosal inflammation in ulcerative colitis and Crohn’s disease. Studies have shown a strong inverse correlation between AF intensity and histological inflammation scores (Osada et al., 2011). Quantitative approaches further improved objectivity by converting AF signals into numerical indices that track disease activity (Moriichi et al., 2015). DL models can automate this quantification, analyzing wide-area endoscopic AF videos to generate “fluorescence activity maps.” Beyond assessing current activity, sequential AF-DL analysis holds promise for predicting relapse. By learning subtle patterns in mucosal healing that are imperceptible to the human eye, these models could identify patients at high risk of flare, allowing for pre-emptive therapy adjustments. AF-DL activity scoring and relapse prediction are emerging tools still under clinical investigation.

### 4.4 Genitourinary system

AF applications in urology are emerging, particularly for non-invasive liquid biopsy and enhanced cystoscopy.

#### 4.4.1 Renal disease and urinary cell AF

A groundbreaking application is the non-invasive assessment of kidney disease via AF analysis of exfoliated renal tubular cells in urine. These cells retain metabolic and structural information. Multispectral AF analysis of these cells can discriminate between patients with preserved and impaired renal function with extremely high accuracy (AUC = 0.99) and can even detect renal fibrosis (AUC = 0.90) (Mahbub et al., 2021). More recently, multispectral AF profiling of urinary exfoliated proximal tubule cells has been investigated in kidney transplant recipients for distinguishing histopathologic causes of graft dysfunction, including acute tubular necrosis, immune rejection, and interstitial fibrosis and tubular atrophy. Machine learning (ML) classifiers facilitate this differentiation, broadening the application scope of “liquid biopsy” beyond native chronic kidney disease (CKD) (Wu et al., 2025). DL can further refine this by classifying cell-level AF patterns associated with specific injury types. This AF-DL urine cytology approach presents a potential paradigm shift, offering a repeatable, non-invasive alternative to renal biopsy for monitoring disease progression and treatment response. Despite exceptional single center accuracy, this approach remains investigational, lacking multicenter validation and standardized protocols.

#### 4.4.2 Bladder cancer

In blue-light cystoscopy (BLC), a hexaminolevulinate-induced porphyrin AF is used to improve the detection of non-muscle-invasive bladder cancer, particularly carcinoma *in situ*. However, manual interpretation is subjective, and inflammatory lesions frequently lead to false-positive results. DL models integrated into BLC systems can effectively mitigate false positives by distinguishing malignant from inflammatory AF patterns. Notably, CNN-based models for bladder malignancy classification yielded excellent diagnostic performance, achieving an AUC of 0.98, 89.7% sensitivity, and 94.0% specificity in image-based validation (Ikeda et al., 2020; Lee et al., 2024). In parallel, real-time AI integration on live cystoscopy video streams is becoming feasible in clinical workflows; a pilot study demonstrated real-time alert overlays during clinic cystoscopy and TURBT, supporting the practicality of AI-augmented cystoscopy rather than purely *post hoc* analysis (Chang et al., 2023). Moreover, dye-free computational strategies are being developed to enhance the accessibility of fluorescence-equivalent contrast. Digital staining techniques can convert conventional white-light cystoscopy (WLC) images into BLC-like representations, achieving a staining accuracy of 80.58% and excellent qualitative and quantitative consistency with ground-truth BLC images. This cost-effective approach broadens clinical adoption in settings where photosensitizer administration is unfeasible or expensive (Chang et al., 2024). BLC is clinically deployed in many centers; DL-enhanced interpretation and digital staining are emerging additions undergoing validation.

### 4.5 Dermatologic and metabolic system

The skin, as the most accessible organ, is ideal for non-invasive AF measurement of cumulative metabolic stress and for oncological margin mapping.

#### 4.5.1 Skin AGE-AF

The accumulation of AGEs in the skin, measured as skin AF (SAF), is a well-validated biomarker for long-term glycemic control and cardiovascular risk in diabetes (Larroumet et al., 2022). In population-based analyses, SAF has also been associated with all-cause and cause-specific mortality, supporting its utility as an integrated marker of cumulative metabolic stress (Boersma et al., 2024). DL is enhancing this field not by analyzing SAF images, but by integrating SAF values with other electronic health record data. Deep survival models can use longitudinal SAF measurements, along with clinical parameters, to more accurately predict individual risk trajectories for diabetic complications, renal decline, or mortality, moving from population-level risk to personalized forecasting. SAF measurement is clinically validated for cardiometabolic risk; DL-enhanced survival modeling is an emerging application.

#### 4.5.2 Dermatological oncology

Multiphoton tomography and FLIM provide high-resolution, label-free imaging of dermal collagen/elastin networks and cellular metabolism in pigmented and non-pigmented skin lesions (Knab et al., 2024). DL models analyze these complex AF/SHG/FLIM datasets and leverage time-resolved bi-exponential AF features from multispectral AF lifetime imaging to distinguish benign nevi from melanoma. Based on both architectural disarray and metabolic alterations, deep neural network models achieve optimal lesion-level classification with 76.84% ± 12.49% sensitivity and 78.29% ± 5.50% specificity (Vasanthakumari et al., 2024). In Mohs micrographic surgery, rapid margin assessment is increasingly being augmented by AI, particularly DL on frozen-section histology, and complementary label-free optical approaches (e.g., AF-Raman) are also being explored for intraoperative detection of residual tumor (Sohn et al., 2021; Boitor et al., 2024). AF-DL for melanoma diagnosis remains investigational, with promising single-center results not yet validated in large multicenter trials.

### 4.6 Nervous system

AF of metabolic cofactors provides a unique window into the energetic state of the brain, both in research and intraoperative settings.

#### 4.6.1 Cerebral metabolic AF

AF in neural tissues largely originates from intrinsic fluorophores linked to cellular metabolism and structural proteins, including reduced nicotinamide adenine dinucleotide, NADH, FAD, lipopigments such as lipofuscin, and protein crosslinks. FLIM, adds a quantitative dimension beyond intensity by capturing lifetime signatures that reflect microenvironment and metabolic binding states, enabling label-free assessment of tissue viability, tumor metabolism, and treatment response. DL can further unlock these weak, heterogeneous signals by denoising, feature learning, and real-time decision support, especially in photon-starved and motion-prone intraoperative scenarios. A practical direction is “fast FLIM,” where DL replaces iterative curve fitting to accelerate lifetime inference and make FLIM more deployable at the bedside and in the operating room (Kapsiani et al., 2025; Shirshin et al., 2022).

For neuro-oncology, optical guidance is increasingly paired with AI to improve tumor visualization, sampling, and margin definition. Although 5-aminolevulinic acid fluorescence is exogenous rather than AF, it represents a mature “fluorescence plus AI” workflow relevant to AF-DL translation. Recent work shows DL can predict intraoperative 5-aminolevulinic acid fluorescence from preoperative MRI, supporting targeted sampling of anaplastic foci and operative planning (Suero Molina et al., 2025). In parallel, AI-enhanced hyperspectral correction and analysis has been explored to improve robustness of intraoperative fluorescence imaging under variable illumination and blood contamination, a key barrier to reliable real-time guidance (Composto et al., 2025).

Beyond dye-guided workflows, label-free approaches are moving closer to intraoperative feasibility. Laser-induced fluorescence and related optical signatures have been studied for intraoperative brain tumor classification, aiming to provide rapid tissue diagnosis without the turnaround time of frozen pathology (Zachem et al., 2025). Mechanistically, AF and FLIM map metabolic reprogramming, NADH pool alterations, and redox shifts that accompany tumor aggressiveness and treatment effects, providing biologically grounded features that DL can learn and generalize (Song et al., 2024).

#### 4.6.2 Intraoperative neurosurgical applications

During brain tumor surgery, especially for gliomas, defining the metabolically active tumor boundary is challenging. Intraoperative AF imaging, AF imaging can leverage endogenous metabolic fluorophores, including NADH and FAD, to reveal spatially heterogeneous metabolic alterations that may correlate with tumor infiltration. DL can process intraoperative optical video streams in real time, generate a tumor probability map, and overlay this map onto the surgeon’s microscopic view to support discrimination of tumor infiltrated brain from edema or normal tissue. This workflow aims to maximize extent of resection while preserving eloquent brain regions, thereby impacting survival and quality of life.

Because many intraoperative glioma workflows rely on fluorescence guidance with 5-aminolevulinic acid (5-ALA), which generates protoporphyrin IX (PpIX), DL has recently been applied to enhance the robustness and interpretability of fluorescence signals under real-time operative conditions, including correction of heterogeneous optical properties and illumination as well as model-based unmixing of hyperspectral fluorescence (Composto et al., 2025; Li Z. et al., 2023). In addition, DL models can predict intraoperative 5-ALA fluorescence based on preoperative MRI in gliomas with low-grade radiographic features, facilitating targeted sampling of anaplastic foci and optimizing surgical planning (Suero Molina et al., 2025; Klint et al., 2025). Finally, label-free endogenous fluorescence spectroscopy combined with machine learning has been validated for rapid intraoperative brain tumor diagnosis, offering a label-free alternative to exogenous dyes (Zachem et al., 2025; Pillar et al., 2024). These AF-DL workflows remain investigational, whereas 5-ALA guidance is clinically established and label-free AF navigation is still in the early translational stage.

### 4.7 Hematology

UV-induced autofluorescence microscopy has also been applied to hematology for label-free peripheral blood smear analysis. Using the smartphone-based autofluorescence microscopy (Smart-AM) system, Huang et al. (2024) realized automated five-part leukocyte differentiation based on intrinsic autofluorescence signals under deep-UV excitation. Their deep learning pipeline combined the Detectron2 framework and pix2pix conditional generative adversarial network (cGAN), delivering an average classification accuracy of 0.982 and an average F1-score of 0.925 for five-part leukocyte identification. This application is emerging, with proof-of-concept accuracy demonstrated but not yet validated across clinical laboratory settings.

## 5 New insights into disease mechanisms revealed by AF-DL integration

The convergence of AF imaging and DL transcends its diagnostic utility, emerging as a powerful discovery platform for probing the fundamental mechanisms of disease initiation and progression. By transforming multidimensional AF data into interpretable biological patterns, this synergy is unveiling pathophysiological insights at an unprecedented scale and resolution.

### 5.1 Metabolic reprogramming and redox remodeling as early disease signatures

A defining advantage of AF based imaging lies in its direct sensitivity to cellular metabolism. Endogenous fluorophores such as reduced nicotinamide adenine dinucleotide, NADH, and FAD act as intrinsic reporters of redox balance, mitochondrial function, and pathway engagement between glycolysis and oxidative phosphorylation. Importantly, these metabolic alterations typically precede overt structural or morphological changes, positioning AF as an upstream indicator of disease initiation rather than a downstream consequence (Song et al., 2024). FLIM, further enriches this metabolic readout by separating free and protein bound fluorophore pools, thereby reflecting enzymatic activity, substrate availability, and microenvironmental stress. These lifetime shifts encode subtle but biologically meaningful differences between adaptive metabolic plasticity and irreversible metabolic collapse. However, the resulting data are high dimensional, spatially heterogeneous, and often obscured by low photon counts and tissue specific optical distortions (Ma et al., 2025).

DL provides the computational machinery required to decode these complex metabolic landscapes. By learning nonlinear relationships across lifetime maps, spectral channels, and spatial context, DL models can identify coherent metabolic phenotypes that correspond to specific disease states, treatment responses, or progression trajectories. A practical driver for translation is that machine learning can accelerate or stabilize lifetime estimation and downstream analysis in photon starved conditions, reducing dependence on slow pixel wise fitting and enabling higher throughput mechanistic mapping (Kapsiani et al., 2025; Gouzou et al., 2024).

### 5.2 Visualizing microenvironmental dynamics and cell-ECM crosstalk

Disease progression is governed not only by intrinsic cellular changes but also by dynamic interactions between cells and their surrounding ECM. AF and multiphoton label free imaging capture key components of this microenvironmental crosstalk, particularly through signals arising from collagen and elastin, whose organization and remodeling reflect mechanical stress, chronic inflammation, fibrosis, and tumor stroma interactions. Spatial remodeling of the ECM alters tissue stiffness, diffusion gradients, and migration pathways, actively shaping cellular behavior rather than serving as a passive scaffold (Chen et al., 2024).

These changes are often heterogeneous and temporally dynamic, forming microenvironmental niches that promote invasion, immune evasion, or fibrotic progression. Conventional histology captures static snapshots but is less suited to quantify spatial organization or dynamic evolution (Li Y. et al., 2025). DL enables systematic analysis of AF derived microenvironmental features by quantifying fiber orientation, density, anisotropy, and spatial coupling to adjacent cellular metabolic states. By integrating structural signals with cellular AF signatures, DL models can reconstruct maps of coordinated cell ECM remodeling, converting microenvironmental dynamics into measurable mechanistic variables (Chen et al., 2024).

### 5.3 Inflammation, field effects, and biochemical heterogeneity

Inflammation introduces profound biochemical heterogeneity into tissues through altered redox metabolism, immune cell infiltration, oxidative stress, and microvascular remodeling. These processes reshape AF landscapes in ways that can overlap with early dysplasia or neoplastic transformation, creating ambiguity when relying on single intensity based features. Hyperspectral AF adds discriminative biochemical content by capturing continuous excitation or emission fingerprints, but the resulting spectral spatial data are complex and high dimensional.

DL is uniquely suited to disentangle overlapping inflammatory and premalignant signals by integrating spectral, lifetime, and spatial information across scales. Rather than treating inflammation as noise, DL models can learn structured patterns that separate transient inflammatory responses from persistent high risk biochemical niches, supporting a field based view of disease biology. A representative example is excitation scanning hyperspectral fluorescence imaging paired with scalable DL frameworks for colorectal cancer detection, illustrating how spectral spatial DL can improve interpretability and robustness in heterogeneous tissue environments (Oswald et al., 2024; Peng et al., 2024).

### 5.4 Neurodegeneration and mitochondrial dysfunction

Neurodegenerative disorders are characterized by early mitochondrial impairment, oxidative stress, and altered redox enzyme engagement, long before neuronal loss becomes morphologically apparent. NADH-FLIM provides a direct window into these early events, capturing changes in mitochondrial efficiency, substrate utilization, and redox buffering capacity at the single cell level. In neuronal models overexpressing Alzheimer disease related proteins, NADH FLIM has shown sensitivity to selective mitochondrial dysfunction, supporting its role as a mechanistically grounded metabolic probe (Niederschweiberer et al., 2021).

However, neural tissues pose challenges for quantitative AF analysis due to low photon budgets, motion, and complex cytoarchitecture. DL addresses these constraints by enabling fit free lifetime estimation, noise robust feature extraction, and automated stratification of heterogeneous neuronal populations. This improves feasibility for mechanistic mapping at scale and supports translation to time constrained experimental and intraoperative settings (Kapsiani et al., 2025; Ma et al., 2025).

### 5.5 Virtual histology and closed loops from mechanism to diagnosis

One of the most transformative outcomes of AF-DL integration is the emergence of virtual histology. By computationally translating label free AF and lifetime data into histology like representations, DL enables reagent free, standardized tissue assessment while preserving underlying biochemical information. A recent study demonstrated DL based virtual hematoxylin and eosin staining from label free AF lifetime images, with clinically oriented evaluation and strong agreement to conventional staining, supporting a practical route to workflow compatible computational pathology (Misra et al., 2026).

Beyond single modality translation, end to end platforms combining hyperspectral AF microscopy with DL based virtual histology have been reported, supporting a broader concept of computational molecular phenotyping from intrinsic fluorescence signatures rather than morphology alone (McNeil et al., 2024). The field is also rapidly consolidating around virtual staining as a general paradigm across label free modalities, with recent reviews outlining clinical translation pathways, validation pitfalls, and standardization needs that are directly relevant to AF-DL (Wong I. H. M. et al., 2024; Latonen et al., 2024).

## 6 AF-DL driven innovations in diagnostic technology

The fusion of AF and DL is not merely enhancing existing diagnostic pathways but is actively forging novel technological paradigms that redefine the boundaries of early disease detection. These innovations span from hardware-software co-design to the creation of entirely new diagnostic readouts.

### 6.1 From qualitative observation to quantitative, standardized biomarkers

A primary innovation of AF-DL integration is converting inherently relative fluorescence intensities into reproducible, clinically actionable biomarkers. In practice, AF signals vary with illumination power, detector gain, tissue hydration, and inter-device optics, which limits cross-center comparability. DL can mitigate these effects via physics-informed normalization, domain adaptation, and uncertainty-aware calibration, enabling harmonized AF feature extraction across devices and sites and facilitating model transportability in real-world workflows (Tian et al., 2021). An example of measurement correction is in ocular AF, where DL reduced cataract-related bias in macular pigment metrics, improving agreement between measurements (Obana et al., 2024).

### 6.2 The rise of real-time, point-of-care diagnostic assistants

**Figure 2:**
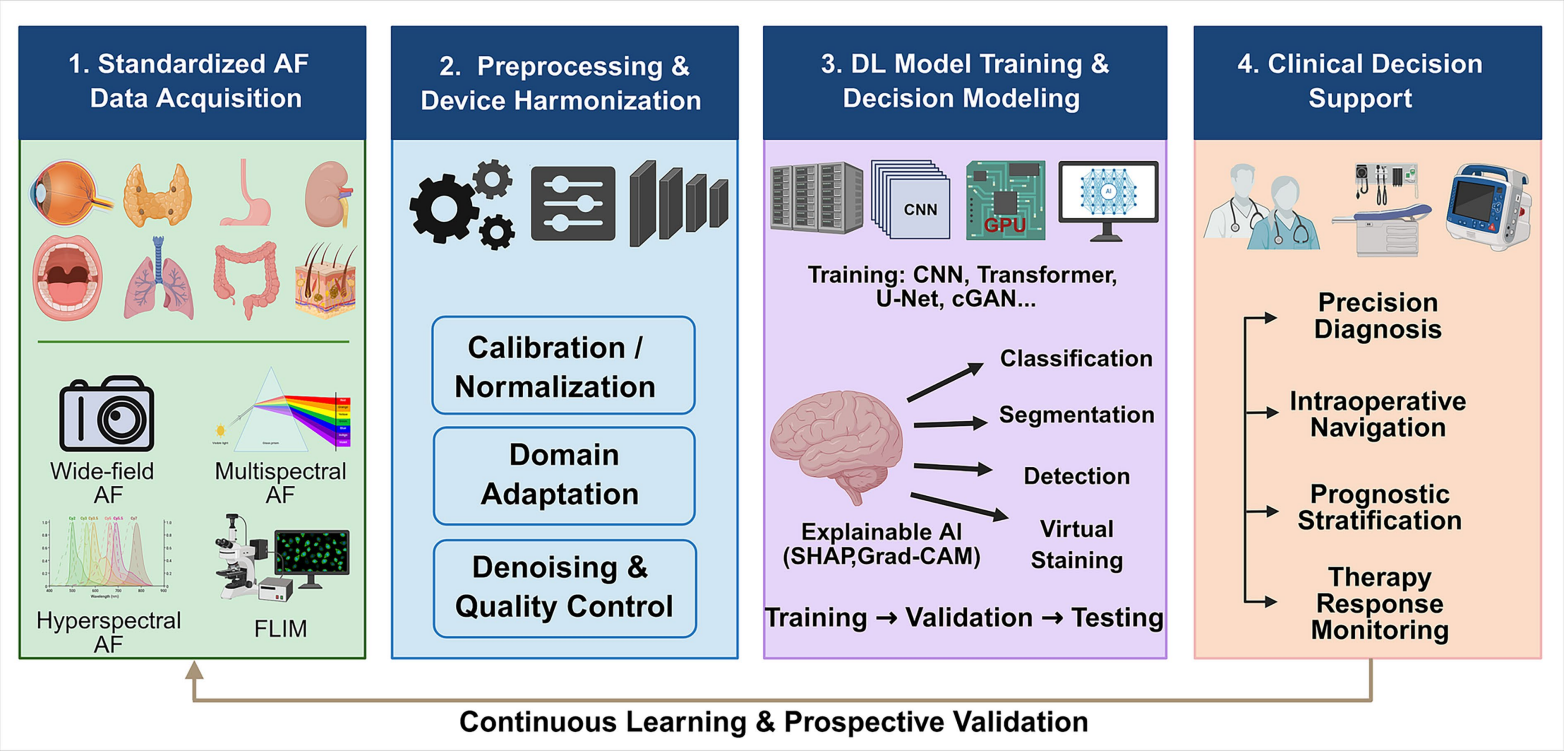
Generalizable workflow for AF-DL integration. The pipeline encompasses four core stages: (1) standardized acquisition of AF data using wide-field, multispectral, hyperspectral, or FLIM modalities; (2) preprocessing for device harmonization, domain adaptation, and quality control; (3) DL-based feature extraction, decision modeling, and explainable AI; and (4) clinical decision outputs spanning precision diagnosis, intraoperative navigation, prognosis, and treatment monitoring. The feedback loop illustrates the need for continuous learning and prospective validation (Created with BioRender).

Miniaturized AF imaging is extending fluorescence-enabled decision support from tertiary centers to community settings. Smartphone-based platforms that combine wide-field white-light and AF imaging with automated risk models can triage oral mucosal lesions and reduce missed referrals, providing an actionable template for scalable AF-DL deployment in resource-limited environments (Mitbander et al., 2025). In wound care, point-of-care bacterial fluorescence imaging leverages endogenous porphyrin and pyoverdine signals to detect clinically significant bacterial burden, support targeted debridement, and enable objective monitoring, creating a natural substrate for DL-based segmentation and outcome prediction (Derwin et al., 2023; Badrie et al., 2025). A generalized end-to-end pipeline illustrating the core stages of AF-DL integration, from standardized data acquisition to clinical decision output, is presented in Figure 2.

### 6.3 Virtual histopathology and label-free phenotyping

In pathology, AF-DL is enabling label-free workflows that shift diagnosis from chemical staining toward computationally generated virtual histology. Hyperspectral AF microscopy paired with DL has demonstrated end-to-end pipelines that acquire unstained tissue AF signatures, generate virtual stains, and support quantitative scoring tasks such as NASH CRN grading with concordance comparable to expert pathologists, highlighting a route to standardized, high-throughput digital pathology (Misra et al., 2026; MacPherson et al., 2024). Beyond H&E, the same paradigm can be extended to multiplexed virtual staining, artifact detection, and slide-level quality control, forming the basis for scalable stain-agnostic pathology pipelines (Li A. et al., 2025).

### 6.4 Closed-loop, adaptive imaging systems

Closed-loop imaging systems represent a frontier direction, in which a low-cost wide-field AF scan functions as a rapid sentinel and a DL controller dynamically triggers higher-resolution or complementary acquisitions only when needed. This paradigm is supported by advances in computational microscopy, where DL reconstructs high-detail fluorescence information from sparse or low-SNR measurements, expanding the feasible field-of-view and improving speed-resolution tradeoffs (Shen et al., 2022; Zhang et al., 2024). In a mature implementation, AF-driven DL gating could reduce data burden, shorten procedure time, and focus clinician attention on biologically suspicious regions, improving workflow efficiency without sacrificing sensitivity.

### 6.5 Multimodal fusion for comprehensive tissue interrogation

Multimodal fusion is likely to be decisive for clinical adoption, because AF provides sensitive biochemical contrast while structural modalities supply depth and morphology. Integrated multimodal platforms combining AF with OCT and other label-free optical contrast mechanisms have been validated for tissue identification and intraoperative surgical navigation. These synergistic systems establish a robust experimental and translational foundation for developing deep learning-based multimodal image fusion architectures (Pan et al., 2021; Shaked et al., 2023). At the algorithmic level, fusion can be performed through feature-level attention mechanisms or late decision ensembles (Huang T. et al., 2025), and future work should prioritize prospective validation, interoperability standards, and human-AI interaction design to prevent overreliance and to maintain clinical trust.

## 7 Applications in therapeutic decision-making, prognosis, and surgical navigation

The clinical value of AF-DL lies in its ability to translate intrinsic optical signatures into decision-relevant information. By capturing metabolic, microenvironmental, and structural features in real time, AF-DL approaches have the potential to inform therapeutic selection, prognostic evaluation, and surgical navigation, addressing clinical questions that are not adequately resolved by morphology-based assessment alone.

### 7.1 Therapeutic stratification and treatment selection enabled by AF-DL

AF-DL can support therapy selection when conventional morphology fails to separate biologically distinct phenotypes with different treatment sensitivities. AF-derived metabolic and microenvironmental features provide functional information related to redox balance, mitochondrial activity, and stromal remodeling. Optical metabolic imaging based on endogenous NADH and flavin signals has demonstrated the ability to characterize treatment-response phenotypes at cellular and tissue levels, linking redox remodeling to therapeutic vulnerability (Shah et al., 2014; Li et al., 2020). DL extends this concept by integrating lifetime, spectral, and spatial context into quantitative phenotype scores. From a decision-making perspective, the most defensible outputs are calibrated response likelihoods or stratification scores that inform choices such as intensifying systemic therapy, adding adjuvant treatment, or favoring surgical intervention, rather than generic claims of personalization.

### 7.2 Prognostic stratification and risk modeling based on AF-DL signature

Prognostic decision-making benefits from AF-DL because intrinsic fluorescence encodes biological heterogeneity that is not captured by size-based staging alone. Metabolic heterogeneity and ECM organization extracted from AF data can be translated into recurrence risk or survival models. Machine learning-based optical metabolic imaging has been associated with clinically relevant outcome prediction, highlighting the prognostic value of intrinsic fluorescence signatures (Walsh et al., 2014). For high-impact clinical translation, prognostic claims should be explicitly linked to endpoints such as progression-free or recurrence-free survival, and evaluated using calibration and decision-curve analyses in addition to discrimination metrics.

### 7.3 Intraoperative surgical navigation and margin assessment using AF-DL

Surgical navigation represents the most direct decision node for AF-DL integration. Intraoperative AF and FLIM enable real-time visualization of tissue metabolism and composition, while DL can generate spatial probability maps indicating tumor infiltration or tissue viability. Label-free fluorescence lifetime and spectroscopic co-registration has been evaluated for intraoperative brain tumor diagnosis, demonstrating functional contrast that complements anatomical guidance during time-critical decisions (Reichert et al., 2021).

DL-based hyperspectral correction and unmixing approaches have further improved robustness of fluorescence-guided surgical workflows under variable illumination conditions, supporting reproducible margin assessment and reducing operator dependence (Black et al., 2024).

### 7.4 AF-DL based therapy response monitoring and adaptive intervention

Response monitoring represents a particularly high-value application of AF-DL integration, as metabolic and redox alterations often precede macroscopic or morphologic changes detectable by conventional imaging. Endogenous AF signals arising from NADH and flavins provide a direct window into cellular metabolic state, allowing early assessment of therapeutic efficacy before size-based response criteria are met.

Recent advances in optical metabolic imaging have demonstrated that therapy-induced shifts in intrinsic fluorescence intensity ratios and fluorescence lifetimes emerge at early treatment time points and correlate with downstream response and resistance phenotypes. These observations have been consolidated in contemporary reviews emphasizing the role of AF-based metabolic imaging as a sensitive biomarker for early therapy response and precision oncology (Engelhardt and Michor, 2021).

Collectively, these studies indicate that AF-DL based response monitoring is most impactful when framed around actionable decision thresholds, such as early treatment escalation, de-escalation, or regimen switching. Future clinical translation will require prospective validation linking early AF-derived response signatures to patient-centered outcomes and predefined adaptive treatment strategies.

### 7.5 Evidence requirements and clinical implementation pathways

Across all decision nodes, the key translational requirement is evidence that AF-DL meaningfully alters clinical management and improves outcomes, rather than merely reproducing retrospective pathology labels. Analyses of fluorescence imaging combined with artificial intelligence in precision cancer surgery emphasize the importance of workflow compatibility, prospective validation, and clinician-interpretable outputs (Cheng et al., 2024; Preziosi et al., 2024). Future studies should prioritize multicenter prospective designs with predefined decision endpoints, clear reporting of failure modes, and assessment of domain shift, particularly for real-time intraoperative applications.

## 8 Challenges, standardization, and clinical translation of AF-DL systems

### 8.1 Signal variability, device dependence, and lack of standardization

A major barrier to the clinical translation of AF and DL systems is the intrinsic variability of AF signals across devices, imaging geometries, and acquisition protocols. Differences in excitation wavelength, detector sensitivity, optical filters, and illumination homogeneity can introduce systematic bias that obscures biological variation, a concern highlighted in recent methodological analyses of *in vivo* FLIM (Ma et al., 2025). Such device dependence is particularly pronounced in AF imaging, where absolute signal intensity is influenced by tissue optical properties rather than fluorophore concentration alone, echoing challenges previously encountered in radiomics harmonization and quantitative pathology. Without standardized calibration strategies and cross-device normalization, AF-based biomarkers risk becoming site-specific rather than disease-specific, a limitation that has been explicitly discussed in the context of fluorescence-guided precision surgery (Cheng et al., 2024).

### 8.2 Algorithmic generalization, bias, and explainability

DL models applied to AF data are vulnerable to overfitting, domain shift, and hidden bias, especially when trained on single-center datasets with limited biological diversity. These risks are well recognized in the broader field of medical artificial intelligence, where inflated performance metrics often fail during external validation (Topol, 2019). In AF imaging, the intertwining of biochemical signals with optical and geometric effects further amplifies the risk of spurious correlations. Explainable artificial intelligence has therefore become a prerequisite rather than an optional feature, with attention mapping and feature attribution methods increasingly used to verify that models rely on biologically plausible AF features instead of acquisition artifacts, as systematically reviewed in medical imaging contexts (Chen H. et al., 2022).

To bridge the gap between computational predictions and clinical biology, the integration of Explainable AI techniques, such as Grad-CAM or SHAP, is becoming indispensable. These tools can provide visual heatmaps that correlate model decisions with specific biological fluorophore signatures (e.g., NADH, FAD, or collagen). This level of transparency is crucial to ensure that DL models are learning from true pathological metabolic changes rather than overfitting to imaging artifacts or background noise, thereby building the necessary clinical trust (Behzad et al., 2024).

### 8.3 Data scale, annotation burden, and ground truth limitations

Robust AF-DL systems require large, well-annotated datasets that link optical signatures to meaningful biological or clinical ground truth. However, acquiring such datasets remains challenging, particularly for intraoperative and endoscopic applications where histopathologic confirmation may be sparse or spatially mismatched. Similar limitations have been described in computational pathology, motivating the development of weakly supervised and multiple-instance learning strategies. Data-efficient learning frameworks that leverage slide-level labels or multimodal co-registration have since been shown to substantially reduce annotation burden while preserving biological interpretability (Nagendran et al., 2020).

### 8.4 Regulatory, ethical, and workflow integration considerations

The integration of AF-DL systems into clinical practice requires alignment with regulatory standards, ethical considerations, and real-world clinical workflows. Unlike retrospective decision-support tools, real-time AF-DL systems may directly influence intraoperative or endoscopic decisions, raising heightened requirements for reliability, latency, and transparency. Guidelines for responsible deployment of DL in healthcare emphasize continuous performance monitoring, dataset shift detection, and post-deployment validation (Esteva et al., 2019). Equally important is workflow integration, as studies of clinical AI adoption consistently show that usability and human-machine interaction strongly determine real-world impact (Sendak et al., 2020).

Beyond algorithmic performance, the successful translation of AF-DL systems must contend with practical, real-world implementation barriers. First, the cost-effectiveness of AF-DL platforms has not been systematically evaluated against current standards of care. While AF imaging offers the advantage of being label-free, the high initial capital cost of multispectral, FLIM, or hyperspectral systems, coupled with the computational infrastructure needed for real-time DL inference, may limit adoption to well-resourced academic centers. Health-economic analyses comparing AF-DL-guided interventions (e.g., in surgical margin assessment or IBD monitoring) against conventional histopathology or watchful waiting are urgently needed to support reimbursement and widespread deployment.

Second, regulatory approval pathways for adaptive or continuously learning DL algorithms remain complex and underdeveloped. Current FDA and CE frameworks are largely designed for locked software as a medical device, whereas AF-DL models intended for continuous learning from new clinical data introduce additional regulatory challenges regarding version control, safety assurance, and post-market surveillance. Early collaboration with regulatory bodies and adherence to emerging guidance on Good Machine Learning Practice will be critical for clinical translation.

Third, clinical adoption is heavily influenced by factors distinct from diagnostic accuracy: computational latency, surgeon or endoscopist ergonomics, and integration with existing electronic health record and operating room ecosystems. If an AF-DL system adds even minimal procedure time or requires frequent manual recalibration, it risks being abandoned despite high analytical performance. Co-design with end-users, usability testing, and workflow simulation should accompany technology development from early stages.

### 8.5 Toward prospective validation and multicenter translation

Moving AF-DL systems from proof-of-concept studies to clinical standards will require prospective, multicenter validation with predefined endpoints and standardized acquisition protocols. Experience from large-scale evaluations of artificial intelligence in medical imaging demonstrates that algorithmic performance often declines when tested across heterogeneous populations and institutions (Liu et al., 2019). Systematic comparisons between artificial intelligence systems and clinicians further underscore the importance of external validation and clinical impact assessment rather than isolated accuracy metrics (Nagendran et al., 2020). For AF-DL, such multicenter efforts are essential not only for regulatory approval but also for disentangling disease-specific AF signatures from site-specific acquisition effects.

## 9 Future directions

AF-DL integration is transitioning from proof-of-concept studies toward clinically scalable diagnostic and decision-support platforms. Several converging technological and conceptual trends are expected to define the next phase of development.

### 9.1 From single-modality AF to multidimensional optical phenotyping

Future AF-based systems will increasingly move beyond single-channel intensity imaging toward multidimensional optical phenotyping that integrates spectral, temporal, and spatial information. Hyperspectral AF and FLIM microscopy provide orthogonal biochemical contrasts that enable separation of overlapping fluorophore contributions and discrimination of free versus protein-bound metabolic states. When combined with DL, these multidimensional signals can be transformed into compact, disease-specific representations that are robust to illumination and geometric variability.

### 9.2 Generalizable models beyond single-disease optimization

Most current AF-DL applications remain optimized for individual organs, devices, or disease entities. A critical future direction is the development of generalizable models that capture shared metabolic and microenvironmental patterns across diseases, enabling transfer learning and rapid adaptation to new clinical tasks. Such approaches may substantially reduce annotation burden and mitigate center-specific bias. Similar challenges of data heterogeneity and task transfer have been widely recognized in artificial intelligence-driven drug toxicity prediction, where models trained across diverse chemical and biological contexts demonstrate improved robustness and generalizability (Lee et al., 2025). Progress in this area will depend on large, heterogeneous datasets and careful validation across institutions rather than continued refinement within isolated single-center cohorts.

Furthermore, overcoming hardware-dependency is a critical challenge for the generalizability of AF-DL models. Variations in excitation light intensity, camera sensor sensitivity, and optical filter bandwidths across different institutions frequently lead to domain shift, where a model trained on one specific system performs poorly on another. To address this, future research must not only prioritize multi-center validation but also focus on developing hardware-independent algorithms and standardized imaging calibration protocols to ensure robust cross-platform performance (Kong and Ban, 2026).

### 9.3 From prediction to biological insight

As AF-DL systems mature, their value will increasingly depend on interpretability rather than raw accuracy. AF signals are intrinsically linked to cellular metabolism and tissue composition, positioning AF-DL as a potential tool for mechanistic discovery rather than black-box prediction. Future research should prioritize models that reveal how metabolic states, stromal remodeling, or treatment-induced shifts are encoded in fluorescence patterns, thereby enabling hypothesis generation and experimental validation. This transition from prediction to biological insight will be essential for clinician trust and scientific impact.

### 9.4 Standardization, prospective validation, and workflow integration

Translation of AF-DL technologies into clinical practice will require standardized acquisition protocols, cross-device calibration strategies, and prospective multicenter validation. For systems that directly influence real-time decisions, such as surgical navigation or early response monitoring, robustness and reliability are paramount. Equally important is seamless integration into clinical workflows, as usability, latency, and human-machine interaction often determine real-world adoption more than algorithmic performance. Addressing these practical constraints will ultimately define whether AF-DL remains a research tool or becomes a clinical standard.

Beyond algorithmic performance, the successful transition from bench to bedside hinges on seamless clinical workflow integration. For real-time applications, such as intraoperative surgical navigation or live endoscopy, AF-DL systems must minimize computational latency to support split-second decision-making without disrupting surgeon ergonomics or prolonging procedure times. Furthermore, navigating the complex regulatory landscape, such as securing FDA or CE approvals for adaptive, AI-driven medical devices, requires rigorous, multi-center prospective trials that validate not only efficacy but also safety and operational feasibility in diverse, real-world clinical environments (Huang J. et al., 2025).

## 10 Conclusion

AF imaging provides a unique, label-free window into tissue metabolism and microenvironmental state, while DL offers the computational capacity to translate this complexity into actionable clinical information. Together, AF-DL integration shifts medical imaging from morphology-based assessment toward functional and biological characterization of disease.

Across diagnostic, prognostic, and intraoperative applications, AF-DL approaches demonstrate particular promise in detecting early and subtle disease-related changes that precede structural alteration. Importantly, the biological grounding of AF distinguishes it from many artificial intelligence pipelines that rely on abstract image features, strengthening its relevance for precision medicine.

The recent clinical demonstration of NIR-II AF for label-free liver malignancy visualization, achieving substantial tumor-to-normal contrast and deeper penetration, exemplifies the rapid evolution of this technology from concept to surgical application. Looking ahead, the impact of AF-DL technologies will depend less on isolated performance benchmarks and more on generalizability, interpretability, and clinical integration. By aligning technological innovation with biological insight and real-world clinical needs, AF-DL has the potential to redefine how disease is detected, monitored, and treated at its most actionable stages.
